# The efficacy of psychological interventions for university students: a systematic review and meta-analysis

**DOI:** 10.1192/j.eurpsy.2023.72

**Published:** 2023-07-19

**Authors:** P. Barnett

**Affiliations:** UCL, London, United Kingdom

## Abstract

**Abstract: Introduction:**

Mental health problems are increasingly prevalent among students, necessitating adequate mental health support both for those who with or at risk of developing a mental health disorder.

**Objectives:**

This systematic review examined the efficacy of psychological interventions delivered to student populations and whether interventions with some form of adaptation to the content or delivery of the intervention for students could improve outcomes compared to interventions which had no such adaptation.

**Methods:**

Randomised controlled trials of interventions for students with or at risk of mental health problems were included. Specific adaptation for students (or whether they utilised a student population as a convenient sample) was recorded. Meta-analyses were conducted and multivariate meta-regressions explored the effect of adaptation on the pooled effect size. Eighty-four studies were included

**Results:**

Promising effects were found for both treatment and preventative interventions for anxiety disorders, depression and eating disorders. PTSD and self-harm data was limited, and did not demonstrate significant effects. Relatively few trials adapted intervention delivery to student-specific concerns, and overall adapted interventions showed no benefit over non-adapted interventions. There was some suggestion that adaptions based on empirical evidence and provision of additional sessions, and transdiagnostic models may yield some benefits

**Conclusions:**

Interventions for students show benefit though uncertainty remains around how best to optimise treatment delivery and content specifically for students. It would be beneficial to understand how intervention content which is specific to underlying mechanisms of problems experienced by students as well as more transdiagnostic approaches could further support recovery and prevention of mental health problems while at university.

**Disclosure of Interest:**

None Declared

